# The Role of Urbanization in Childhood Obesity

**DOI:** 10.4274/jcrpe.1984

**Published:** 2015-08-31

**Authors:** Özgür Pirgon, Nagehan Aslan

**Affiliations:** 1 Süleyman Demirel University Faculty of Medicine, Department of Pediatric Endocrinology, Isparta, Turkey; 2 Süleyman Demirel University Faculty of Medicine, Department of Pediatrics, Isparta, Turkey

**Keywords:** obesity, childhood, prevention, environmental factors, urban environment

## Abstract

Obesity is becoming the most frequently diagnosed chronic disease in many countries affecting all age groups and specifically the pediatric population. To date, most approaches have focused on changing the behavior of individuals with respect to diet and exercise. Almost all researchers agree that prevention could be the key strategy for controlling the current epidemic of obesity. Prevention may be achieved by changes in lifestyle through a variety of interventions targeting the urban environment, physical activity, time spent watching television and playing computer games and consumption of carbonated drinks. However, as yet, these strategies seem to have had little impact on the growing increase of the obesity epidemic. In this article, we aimed to discuss the effect of rapid urbanization on childhood obesity and to suggest solutions to this problem.

## INTRODUCTION

Obesity, which is defined as an increase in the amount of body fat, occurs as a result of an imbalance between energy intake and output. Besides causing health and social problems, obesity leads to approximately 30 000 early deaths per year and continues to be an increasing public health problem both in Turkey and around the world (1). For example, in the United Kingdom (UK), despite a reduction in food calorie intake in the last 30 years, the prevalence of obesity in the last 20 years has increased three-fold and the annual costs of healthcare related to obesity have reached approximately 3.3-3.7 billion sterling (5.5 billion US dollars) (2,3).

Although prevalence of obesity is increased in all age groups, this increase is encountered more frequently in ages at which there is more rapid fat storage physiologically. In a previous study, it was determined that 26-41% of obese pre-school children and 42-63% of overweight school children were obese as adults (4). This study is important in that it shows the importance of the health infrastructure in childhood for the health status of adults. Obesity is thought to be more dangerous when it starts before the age of 5 years or after the age of 15. It has been shown that the dramatic increase in obesity in children which has occurred in the last three decades can lead to depression and various diseases such as asthma, fatty liver, sleep apnea, hypertension, orthopedic problems and type 2 diabetes (5,6).

The aim of this paper is to review the role that an obesogenic environment including factors such as city planning, school-life and neighborhood relations can play in the recent increase in the rates of childhood overweight/obesity.

## Obesogenic Environment

Several factors such as genetic tendency, environmental and cultural structure of the society and socioeconomic level may lead to childhood obesity. However, the relative weight of each of these factors labelled as ‘obesogenic causes’ in the development of obesity has not yet been clarified (6). Although genetic tendency is important, the environmental factors are often accepted as the causes of obesity. Genetic and environmental factors separately or together lead to an increase in weight.

The concept of obesogenic environment has been first reported by Swinburn in 1999 as “excessive weight not as the result of a single cause but as the significant effect of environment on nutrition and physical activity”. According to Swinburn, obesity in individuals and society can be prevented with a reduction in obesogenic factors (7). Obesogenic environment is a complex and multi-dimensional concept. After Swinburn, obesogenic environment was again defined by Hill, Wyatt, Reed and Peters in 2003 explicitly as an environment leading to excessive calorie intake and sedentary lifestyle of an individual (8).

**Figure 1:**
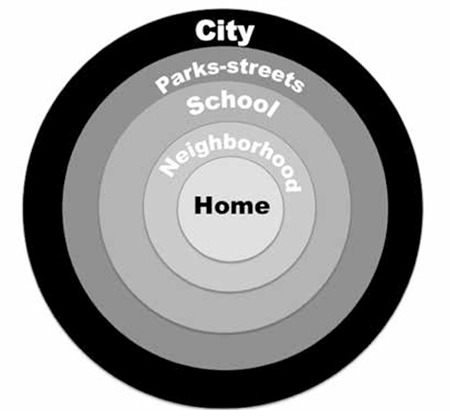
The Obesegenic Environment: the sum of influences which affect the life of children (illustrated by Özgür Pirgon)

Despite ongoing attempts to reduce the prevalence of obesity with different measures such as pharmacological treatments, diets, education and methods aiming to alter behavior, these have so far remained at a limited level of success. Generally, the most important steps in the prevention of obesity are diet and physical activity. However, several factors affect these. The residential environment, cultural structure, social relationships and extent of surrounding areas have been determined as the most important environmental factors (Figure 1).

Together with the known effects of residential environment on health, there are also effects on intellect, mental development, as well as on physical and emotional structure. Most studies have shown that childhood obesity is related to the environment around the home of the child (9,10). Obese individuals are found more often in communities with low economic structure and insufficient socialization/play areas as they do not have an active lifestyle. In contrast, if there are walking areas and parks where physical activity and exercises can be undertaken in the residential area of a child, it is easier for the child to lose weight. The extent of recreational areas close to the safety of the city provides the opportunity for an individual to exercise comfortably (11). In a Turkish society, security concerns restrict the activities of children, particularly of girls and this situation leads to increased weight together with social problems (5).

In the studies by Swinburn, environmental factors of the micro-environment (school, workplace, home, neighborhood) and the macro-environment (education and health systems, government policies, societal involvement, cultural belief structure) were defined (7). The effectiveness of a regulation of the environment imposed by the State in reducing the prevalence of obesity is not known, but it is thought that it could be useful in facilitating societal effects and individual effects. Environmental requirements in the prevention of childhood obesity are listed in Table 1.

## Healthy Cities

Urbanization is one of the most important actors of an obesogenic environment. Studies investigating the causes for the increase in frequency of obesity have identified a strong correlation between obesity and urbanization and also between obesity and physical activity and chronic diseases. An inadequate social environment and buildings not suitable for walking around push children into an inactive lifestyle. Therefore, in urban planning, an environment should be provided taking public health and the risk of obesity into account.

**Figure 2:**
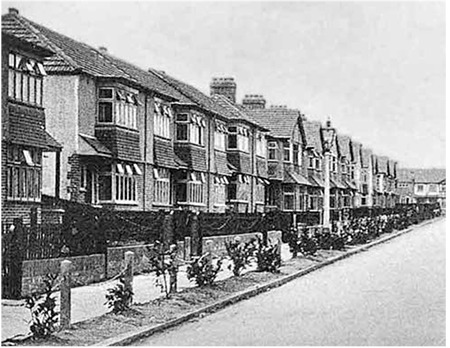
Typical row of 1930s-built semi-detached English suburban houses (Orpington Gardens, N18), photographed in the 1930s

During the industrialization of the UK and the United States of America (USA), modern cities were established starting from the 19th century and while these buildings and mass dwellings made life easier for people, there were fewer play areas for children and it was not thought that this could lead to an increase in the frequency of obesity in society. While suburban residences were a priority in 1930s England (Figure 2), in the 1960s a strong relationship was determined between the health of an individual and the place of residence and in later years, residential projects were realized with better planning in terms of health (12). This engendered the necessity of extensive living areas such as the width of the street and houses having a garden or terrace (13).

While the treatment for obesity in some countries has mostly consisted of various medical therapies and diets, developed countries have focused on the construction of long-term life plans. After the 1980s, the concept of ‘Healthy Cities’ was introduced to reconstruct cities with an emphasis on public health (14). Besides making life easier, these cities provide important facilities in terms of health by including park areas, extensive walking and cycling pathways, as well as areas for sporting activities.

The introduction of a project entitled ‘Healthy Cities’ by the World Health Organization in 1987 led to a recognition of the importance of social areas and of organizing an environment suitable for physical activity by all European countries (8,13). In the USA, a new urbanization period was started with the founding of the two groups, namely, the ‘Healthy Society Movement’ and the ‘Healthy Cities and Society’ (15). As a result, safe walking pathways were built, businesses were established which could be reached by pedestrians and public transport was made more attractive by lowering prices. In the UK, a government policy of priority for pedestrians in cities was introduced to reduce car journeys and give more importance to pedestrians.

## Urbanization and Obesity

**Figure 3:**
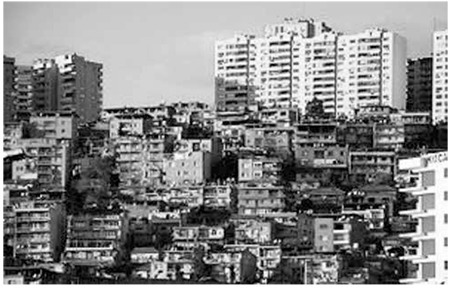
Rapid urbanization (İzmir, Turkey) in the late 1990. No suitable walking and play areas

Recent studies suggest that rapid urbanization accounts for significant shifts in dietary patterns and for physical activity levels that tend to increase risks for obesity in children (16). Since the early 1980s, Turkey has been going through a rapid urbanization process at a pace beyond the world average (Figure 3). In Turkey, the urbanization rate has shown a sudden increase in certain cities because of the migration from rural to urban areas beginning in the 1950’s. Thus, the ratio of the urban population to the total population in Turkey which was 18.7% in 1950, increased to 25.9% in 1960 and to 45.4% in 1980. Highly urbanized areas have led to diminished access to sporting activities and other means of physical exercise. As there are no suitable walking and play areas, families living in inner city areas do not send their children outside but prefer them to stay indoors playing on a computer or watching television. After a certain age, the child becomes accustomed to an inactive lifestyle and having adopted this lifestyle, finds it difficult to socialize. Lack of sufficient areas suitable for walking and cycling when urban planning has been reported to be related to obesity (17). We suggest that in urbanization, sufficient recreational areas must be provided; historical areas should be within reach by walking; high quality, safe-walking pathways should be made available and thus people encouraged to walk and cycle. In recent years, there is some effort for construction of walking and cycle paths in many Turkish cities.

In studies conducted in the USA and Australia, traditional neighbor relationships have been found to be one of the factors increasing the frequency of obesity (18,19). For example, houses very close to one another lead to shorter walking routes. In a study conducted in San Diego, in areas where the walking distance in the neighborhood between acquaintances was far enough to take more than 1 hour, the prevalence of overweight was reported as 35% and in areas with shorter distances as 60% (19). In a recent review, Black and Macinko (20) found evidence from North America, Britain and Australia suggesting that lower socio-economic status neighborhoods and those with larger minority populations have greater exposure to fast-food restaurants and fewer healthy food choices. In a study conducted in Atlanta, USA, a strong relationship was determined between body mass index and urbanization. On housing estates with close neighbors, the frequency of obesity was determined as 23% and where there were longer distances, it was 13% (21). According to the region, as population density decreases, obesity decreases. In a study in New York on 13.367 subjects, a relationship was determined between body mass index and building structures (density, proximity to stations, distance to bus stops, width of streets and connections to one another) (22). Thus, the urban fabric was defined as the most important environmental factor in the prevention of obesity due to its effect on people’s desire and freedom to go outside. In addition, the study also emphasized that not owning a car as well as having parking difficulties were also reasons that reduced weight gain.

## School Life in Cities

Many countries have nutrition guidelines for school meals. Although many attempts have been made to improve the environmental factors which have a negative effect on child and adolescent health in schools, a sufficient effect has not been obtained as yet. Especially in adolescence, the formation of behavior in areas such as physical activity, food habits and harmful habits is very often affected by peers at school. Most studies in this field have aimed to create an awareness of correct nutrition to the students as well as to the school administration (23,24,25). Changes in school lunch programs have resulted in significant improvements in diet and eating behavior (26).

In 2005, many schools in Belgium, Germany, France and Sweden banned vending machines (the sale of candy and soft drinks) in all of the city primary schools. If high-fat snacks are offered and sold to students at school through vending machines or cafeterias, they will displace fruits and vegetables. To give positive messages about healthy nutrition, education programs have been developed including shopping of healthy foods, preventing the sales of high calorie food and drinks in school canteens and shops around the school and stressing the importance of physical activity. These studies have had a positive effect on children’s food behavior as well as their weight, mental health and general lifestyle.

In a study carried out in Israel where young people met celebrities as active participants in an educational entertainment, significant improvements were achieved in the attitudes of the participating young people towards physical activity and diet (27). In contrast, there are studies showing that school-based obesity programs did not change nutrition at school or physical activity (28,29,30). In a study by Simsek et al (31) on 1510 school-age children (6-17 years) in Ankara, it was reported that obesity was a significant problem in schoolchildren, particularly in adolescence and it was shown that in addition to genetic predisposition, nutritional habits and insufficient physical activity could also be significant factors. Oztora et al (32) reported a statistically significant increase in the frequency of risk factors such as unhealthy eating habits and spending long hours on watching TV in obese school children. Similarly, Akac et al (33) observed that obesity in primary school children correlated with television viewing and computer time exceeding 4 hours.

In this context, school healthcare services must include education of the children on correct nutrition. Also, the food provided in school canteens must be mandated to sell healthy foods. The educational programs should emphasize the importance of prevention of becoming overweight and an awareness should be created at an early age to increase physical activity and prevent obesity in children.

In conclusion, recent studies on childhood obesity have shown the importance of specific planning for children in the management of obesity and also the need for assessment of the environmental factors causing obesity in the region lived in and improvement in this area. It has been shown that allotting extensive areas and providing facilities for exercise lead to increase in physical activity. Once obesity has developed, treatment is difficult and obesity which develops in childhood and adolescence tends to continue into adulthood. The health problems that an obese child will encounter in the future will reduce his/her quality of life. As adolescent health is reflected in adulthood, it is necessary to manage it effectively in this period. Intervention in the current epidemic of obesity requires individual behavior changes, but to prevent obesity, it is important that these changes are supported by a societal approach.

## Figures and Tables

**Table 1:**
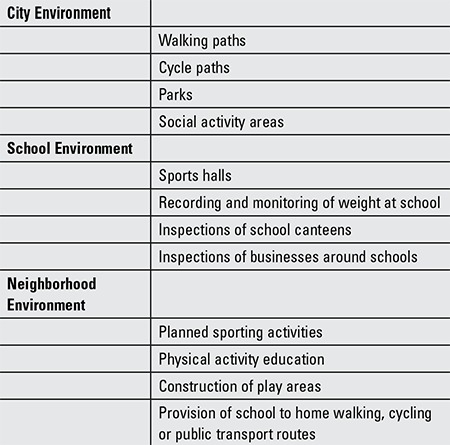
Environmental requirements in the prevention of childhood obesity
